# A time-restricted feeding intervention in children and adolescents with obesity: The TRansForm study protocol

**DOI:** 10.3389/fnut.2022.1026694

**Published:** 2022-10-26

**Authors:** Paula Molina-Giraldo, Serafin Murillo, Laura Meis, Oscar Sans, Montse Amat-Bou, Marina Llobet, Josep C. Jimenez-Chillaron, Marta Ramon-Krauel, Carles Lerin

**Affiliations:** ^1^Department of Endocrinology, Institut de Recerca Sant Joan de Déu, Hospital Sant Joan de Déu, Barcelona, Spain; ^2^Pediatric Sleep Unit, Neurophysiology Division, Department of Neurology, Hospital Sant Joan de Déu, Barcelona, Spain; ^3^Department of Physiological Sciences, School of Medicine, University of Barcelona, L’Hospitalet de Llobregat, Spain

**Keywords:** time-restricted feeding (TRF), childhood obesity, circadian rhythm, gut microbiota, actigraphy

## Abstract

Obesity during childhood is of special concern as adiposity is typically tracked into adult life and it constitutes a major risk factor for future obesity and associated metabolic disorders. Recent studies indicate that time-restricted feeding (TRF) interventions may provide a promising strategy for obesity treatment. However, TRF interventions have only been tested in adult subjects. This study aims to determine both short- and long-term effects of a TRF intervention in children and adolescents with obesity. We will also investigate potential mechanisms mediating the response to the intervention, including the circadian rhythm and the gut microbiota composition. We have designed a randomized-controlled parallel-group clinical study in which children and adolescents (age range 8–18 year-old) with obesity will be subjected to time-restricted eating or no time restrictions for 2 months. Follow-up visits will allow for long-term effect assessments. We will measure anthropometric (BMI, body composition) and metabolic parameters (glucose and lipid metabolism), indicators of the circadian rhythm, and gut microbiota composition will be analyzed. This study will (1) determine safety and effectiveness of the TRF intervention in children and adolescents; (2) assess its long-term effects; and (3) evaluate potential mechanisms involved in the response to the intervention.

**Clinical trial registration:**

[www.ClinicalTrials.gov], identifier [NCT05174871].

## Introduction

The worldwide prevalence of obesity has steadily increased during the last decades (1). Obesity in children is of particular concern, as excessive weight gained during childhood can be tracked into later life: approximately 80% of children with overweight or obesity will stay obese as adults, thereby increasing risk for associated metabolic disorders including type 2 diabetes and cardiovascular disease (2–7). Importantly, improving obesity status during childhood and adolescence is associated with reduced risk for obesity and co-morbidities during adulthood (7). Lifestyle interventions aimed at increasing physical exercise and improving diet quality are the main current obesity therapeutic strategy. However, these interventions have proven challenging to implement at the population level and results are typically insufficient, especially regarding long-term maintenance of the beneficial effects. Therefore, developing more efficient strategies to tackle childhood obesity is a public health imperative.

Time-restricted feeding (TRF) interventions have recently emerged as a promising strategy for weight loss and metabolic health improvements. This strategy consists of restricting the hours for feeding to a specific interval during the day. An increasing number of studies have examined the effects of TRF in adult subjects [for a review of studies see (8)]. These studies are very heterogeneous as they show different (1) length for the TRF (ranging from 4 to 13 h, with a majority of studies between 8 and 10 h); (2) time of day for the restriction (breakfast, lunch, or dinner); (3) duration of the intervention (from 4 days to 16 weeks, being 8–12 weeks the most common time period); and (4) target population (from athletes to adults with obesity) (8). Despite this heterogeneity, the studies generally report significant improvements in BMI, lipid metabolism, and glucose homeostasis (8, 9). However, most interventions targeted adult subjects and there is a lack of data regarding the effects on younger individuals.

**FIGURE 1:**
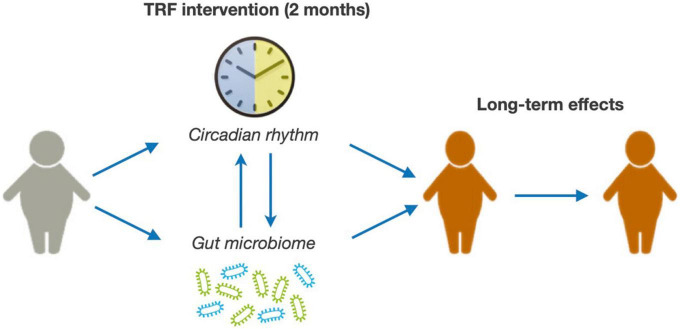
Summary of the study hypothesis.

The main objective of this clinical study is to evaluate the safety and effectiveness of a TRF intervention in children and adolescents with obesity and determine whether the potential benefits of the intervention are maintained over time (Figure 1). Furthermore, we will explore potential mechanisms mediating TRF effects, specifically focusing on the role of the circadian rhythm and the gut microbiome (Figure 1). For this purpose, we have designed a randomized clinical study in which children and adolescents with ages between 8- and 18-year-old with obesity are subjected to a 2-month TRF intervention and followed-up for 24 months. We will measure anthropometric parameters, adiposity, glucose metabolism, and the lipid profile.

## Methods and analysis

### Experimental design and study setting

This is a parallel-group randomized-controlled clinical study performed at the Endocrinology department of Sant Joan de Déu Children’s Hospital, a tertiary care center located in Barcelona (Spain). Recruitment started in January 2022 and is expected to end in December 2022. Candidates undergo the study screening on their regular visits at the hospital and are informed about the study. Whether they agree to participate, parents or legal guardians sign the informed consent, and participants over 12 years of age sign the informed assent document as well. Participants are then randomly assigned to two groups for a 2-month intervention:

- Control group: this is an active control group with the standard obesity treatment protocol at the hospital and no feeding time restrictions.

- TRF group: in addition to the same standard treatment protocol, participants in this group will be subjected to TRF (16 h fast and 8 h feed, 6 days per week).

The study is in accordance with the Helsinki Declaration of 1975 (revised in 2008), has been approved by the local Ethical Committee (ID: PIC-130-219), and was registered at ClinicalTrials.gov (ID: NCT05174871) prior to the start of recruitment.

### Recruitment and eligibility criteria

Participants are recruited from the obesity clinical program at Hospital Sant Joan de Déu based on the eligibility criteria stated below. Approximately 50% of patients with obesity are female so we expect a similar percentage of participants in the study. The study is introduced to the families by the pediatrician and those interested in participating are scheduled for a screening visit at the hospital where they sign the informed and assent consent documents. At the screening visit, 2 weeks before the baseline visit (time 0), participants are provided with an ActTrust 2 actigraph that they wear until the next visit, a fecal sample collection kit, and instructions to fill up a 4-day food registry. At the baseline visit, participants return the actigraph and provide the fecal sample and the food registry.

Participants are selected based on the following criteria:

- Inclusion criteria: age between 8 and 18 years and obesity, defined as BMI-SDS > 2 standard deviations based on World Health Organization growth charts (WHO).

- Exclusion criteria: bariatric surgery; spontaneous TRF for more than 12 h per day; diabetes with insulin treatment; pregnancy; intellectual disability; prescription of drugs for appetite or weight loss (or changes in dose) during the 3 months prior to recruitment.

### Intervention

All participants receive the standard obesity treatment protocol at the Hospital, reported elsewhere (10, 11). This protocol consists of a family-targeted behavioral intervention provided by pediatric endocrinologists and dietitians aimed at implementing healthy dietary habits and increasing physical activity (10, 11). Briefly, dietary recommendations are based on the Mediterranean diet following our local guidelines. We use the Harvard plate method (12) as a tool to improve portion size and balance the meals aiming to reduce overall calorie consumption. Furthermore, we advise participants to limit sedentary activities and screen time, as well as to get active in their daily activities. Finally, we recommend a minimum of 60 min of moderate intensity physical activity per day, according to WHO guidelines.

In addition to the standard protocol, participants randomized into the TRF group have their feeding time restricted to 8 h per day for 6 days per week, with 1 day with no time restrictions. Only water and infusions with no sugar or artificial sweeteners are allowed during the fasting period. To facilitate adherence to the intervention, participants and families are allowed to decide the no-restrictions day as well as the 8-h time interval that best fits their daily schedules; however, this interval is always between 10:00 and 22:00 (for instance, a family can choose 10:00–18:00, while another one can choose 14:00–22:00). Participants use a calendar page to report the days they completely adhere to the intervention (8 h of feeding time), partially adhere (for instance, 10 h instead of 8 h), or did not adhere (12 h or more). A phone call at the third week of intervention and a presential visit at the hospital at week 5 allow for adherence monitoring and improvement. Potential adverse events are evaluated at the 3-week phone call, 5-week, and 2-month visit, and specifically include headache, dizziness, diarrhea, and constipation, as well as any other health-related problem participants might report. Furthermore, a physical medical examination is performed by the pediatrician at the 5-week and 2-month visit. Other parameters considered for safety assessment include anthropometric measurements, hepatic and renal function, glucose and lipid metabolism, and mental health symptoms assessed at all time-points (see section “Outcomes” for details).

### Study visits

**FIGURE 2:**
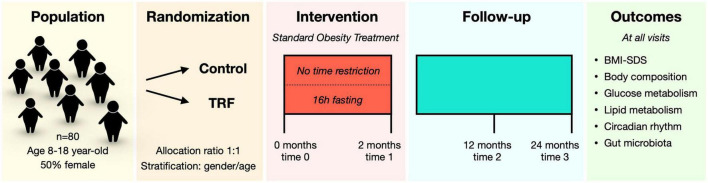
Schematic representation of study design.

To facilitate logistics of the study, participants are organized in groups with one group starting the intervention every month. Thus, the study is being performed with monthly staggered visits with a maximum of 2 overlapping groups during the 2-month intervention. The intervention visits take place at baseline (time 0) and at the end of the intervention (time 1) (Figure 2). Follow-up visits are scheduled at 12 months (time 2) and 24 months (time 3), and will allow for assessing the long-term effects of the intervention (Figure 2).

### Outcomes

Study outcomes are measured at all-time points (0, 2, 12, and 24 months). The change from baseline to each time-point will be evaluated. The study outcomes are the following:

(i) Primary outcome

*Change in BMI-SDS from baseline*. Weight and height are monitored at all-time points with an electronic scale and stadiometer and combined to calculate BMI-SDS based on the WHO growth charts. Participants will wear minimal clothing during the height and weight measurements.

(ii) Secondary outcomes

*Change in metabolic parameters*. A fasting blood test is performed to analyze glucose metabolism parameters (glucose, insulin, HOMA-IR, and HbA1c levels) and the lipid profile (triglycerides as well as total and LDL/HDL-cholesterol). Circulating hepatic markers GGT, ALT, and ASP, which will allow us to estimate the effects of the intervention in the hepatic tissue, are also quantified. Diastolic and systolic blood pressure is also monitored at each visit.

*Change in body composition*. Body composition is assessed by bioimpedance with a Tanita MC-780 BC instrument, available at the Hospital’s Endocrinology department. We are monitoring the absolute amount and percentage of both total body fat and lean mass as well as abdominal fat mass at all-time points.

*Nutritional analysis*. Four-day food registries are used to quantify daily energy intake as well as dietary macro- and micronutrient composition, providing a context for the potential changes induced by the intervention. The nutritionist reviews the registries with the participants and families at each visit to ensure accurate reporting of portion sizes, cooking methods, ingredients, and intake times. The dietary analysis is performed with DIAL software (Alce Ingenierìa, Spain), which provides daily energy consumption, as well as macro- and micro-nutrient intake.

*The children’s depression inventory*. This is a 27-item scale that is used to evaluate depression symptoms in children and adolescents. It evaluates baseline depression and any potential changes induced by the intervention.

*Circadian rhythm and sleep analysis*. Actigraphy is used to accurately monitor circadian rhythm. Participants are instructed to wear an ActTrust 2 actigraph (Condor Instruments, Brazil) for at least 10 days before each visit. The ActTrust 2 is a wearable wrist band specifically designed to make accurate continuous measurements of activity, light, and temperature. The data collected is used to measure sleep and wakefulness patterns as well as parameters directly related to circadian rhythm, including temperature, activity, and ambient light variation. Actigraphy data will be analyzed with the ActStudio^®^ software (Condor Instruments, Brazil). This software transforms the collected data into the parameters that conform rhythmic behavior, as defined by a wave curve. These parameters include amplitude, acrophase, M10 (daily 10 h of maximum activity) and L5 (daily 5 h of minimum activity), as well as interdaily stability and intradaily variability. In addition, participants answer the Sleep Disturbance Scale for Children (SDSC) (13) and Pediatric sleep questionnaire (PSQ) (14) at every visit to screen for sleep quality and potential problems.

*Fecal microbiota analysis*. Bacterial DNA will be isolated from stool samples and microbiota composition will be determined by 16S-sequencing. With this analysis, we will evaluate intervention-induced changes in the intestinal microbiota composition, and assess whether these potential changes are maintained over time.

### Sample size

With no data on TRF interventions in subjects within our age range, an exact calculation of sample size is not feasible. To estimate sample size, we relayed on our data from more than 150 patients with obesity (8–18 years of age) at the Hospital’s Endocrinology department that have undergone the standard obesity treatment protocol. This group would be equivalent to the Control group. The standard deviation of BMI-SDS change over a period of 2 months during a lifestyle intervention was 0.35 units. Based on these data, with type I error of 0.05 and power set at 80%, we will need 64 subjects to detect a difference of 0.25 units in BMI-SDS change between groups. To account for a potential high drop-out rate, we aim to recruit a total of 80 subjects, anticipating up to a 20% participant loss.

### Randomization

Participants are randomly allocated into Control and TRF groups with an allocation ratio of 1:1. Randomization is performed in blocks (block size of 4) stratifying by gender and age (younger/older than 12 years of age) to allow for a balanced number of participants in each group. Randomization lists were obtained from computer-generated sequences. Participant randomization is implemented in REDCap (Research Electronic Data Capture).

### Data management and analysis

Study data are collected, managed, and stored using REDCap tools hosted at Hospital Sant Joan de Déu (15, 16). REDCap is a secure, web-based software platform designed to support data capture for research studies. To assess the comparability of the two groups, continuous variables will be compared using Student’s *t*-test or non-parametric tests for those with a non-normal distribution. Categorical variables will be assessed with the Chi-square test. Multivariate linear and logistic regression models will be used to analyze the effects of the TRF intervention in comparison with the Control group. These methods will allow for adjustment by potential confounding variables, including gender, age, baseline BMI-SDS, and pubertal status. Furthermore, the design of the study allows for secondary analyses. Specifically, we will carefully examine whether (1) gender; and (2) pubertal status are determinants of the short- and long-term TRF-induced effects by analyzing the different data subsets. Statistical calculations will be performed in JMP v16 software (SAS Institute Inc., Cary, NC).

## Discussion

Given the current status of the obesity epidemic and the fact that most children with obesity will track adiposity into adulthood, developing novel and more efficient strategies to tackle childhood obesity is an urgent need. TRF interventions are emerging as a promising strategy to treat obesity and improve metabolic status in adult subjects (8). However, very limited data are available describing this type of interventions in children and adolescents. A study with forty-five adolescents (age range 12–17) with intermittent energy restriction showed metabolic improvements (17). The intervention consisted in a very low-calorie diet for 3 days per week and achieved reductions in BMI and body fat as well as in several cardiovascular risk markers. Furthermore, a recent report showed no effects of a TRF intervention in a study with fifty adolescents (age range 14–18) (18). This intervention implemented a feed time restriction of 8 h, similar to our study; however, it differed in the length of the intervention (3 months) and the number of days per week with time restrictions (5 days per week). To our knowledge, the TRansForm study is the first to test a TRF intervention in children with obesity as young as 8 years of age. This study will evaluate the safety and efficacy of this intervention in this pediatric population with obesity.

The clinical study will also provide valuable information on circadian rhythm patterns in children and adolescences with obesity. Circadian rhythms are physiological processes that primarily respond to light and dark cycles [for review see (19)]. These are controlled by an internal circadian clock and are crucial in multiple important functions in mammals, including sleep patterns, eating habits, hormone release, and body temperature (19, 20). Notably, TRF interventions seem to exert their beneficial effects, at least in part, through modulating the circadian rhythm (21). Indeed, peripheral tissues, including liver or white adipose tissue, set their endogenous rhythmicity in response not only to light-dark cycle cues (through hypothalamic signals) but also to nutritional cues (22, 23). Hence, restrictions in feeding times modulate the rhythmic behavior of peripheral tissues, thereby impacting metabolic outcomes.

Genetic evidence in animal models supports a causal role of circadian rhythm disruptions in inducing metabolic derangements, including hyperglycemia, hyperinsulinemia, impaired lipid metabolism, and hepatic steatosis (24–28). In agreement, alterations in circadian systems may contribute to obesity-related complications in humans, including T2D and hypertension (29–36). In recent years, several studies have unveiled a functional interaction between circadian rhythms and the gut microbiome that may influence host metabolism and glucose homeostasis (37–40). The gut microbiome itself has been shown to be an important player in the development of obesity and T2D (41–43). Despite all these data, further evidence is needed to determine whether this gut microbiota-circadian clock axis plays a role in the development of obesity and metabolic diseases.

Limitations of this study include a potential high drop-out rate in young children; while studies in adults and adolescents generally show high adherence to the intervention, no data are available for young children. In these regards, we have anticipated a 20% drop-out rate when calculating sample size. Second, all participants will be from the same geographical area (mostly Barcelona’s metropolitan area); therefore, caution will need to be exerted when extrapolating the results to other populations. Third, while the study will be powered for the analysis of the main outcome, secondary analysis with data subsets (namely, gender and pubertal status) may be underpowered. Finally, because of ethical reasons we cannot include a group of children and adolescent with obesity with no intervention as all patients receive treatment at the hospital.

In summary, this study will assess the safety and efficacy of a TRF intervention in children and adolescents with obesity and determine the long-term effects of the intervention. Positive results from this study will provide new evidence-based tools to endocrinologists, dieticians, and other practitioners to tackle childhood obesity.

## Ethics statement

The studies involving human participants were reviewed and approved by Comitè d’Ètica d’Investigació amb medicaments (CEIm), Hospital Sant Joan de Déu, University of Barcelona (Spain). Written informed consent to participate in this study was provided by the participants’ legal guardian/next of kin.

## Author contributions

CL, MR-K, and JJ-C: study conceptualization. PM-G, LM, OS, and MA-B: design of recruitment and study visits. SM and ML: design nutrition intervention. CL: writing—original draft. All authors writing—review and editing.
